# The choice of statistical methods for comparisons of dosimetric data in radiotherapy

**DOI:** 10.1186/1748-717X-9-205

**Published:** 2014-09-18

**Authors:** Abdulhamid Chaikh, Jean-Yves Giraud, Emmanuel Perrin, Jean-Pierre Bresciani, Jacques Balosso

**Affiliations:** University Joseph Fourier, Grenoble, France; Department of Radiation Oncology and Medical physics, Grenoble University Hospital, Grenoble, France; University Claude Bernard Lyon 1, Villeurbanne, France; Department of Medicine, University of Fribourg, Fribourg, Switzerland; LPNC, CNRS & Grenoble-Alpes University, Grenoble, France

**Keywords:** Statistical methods, Dose, Radiotherapy

## Abstract

**Purpose:**

Novel irradiation techniques are continuously introduced in radiotherapy to optimize the accuracy, the security and the clinical outcome of treatments. These changes could raise the question of discontinuity in dosimetric presentation and the subsequent need for practice adjustments in case of significant modifications. This study proposes a comprehensive approach to compare different techniques and tests whether their respective dose calculation algorithms give rise to statistically significant differences in the treatment doses for the patient.

**Methods:**

Statistical investigation principles are presented in the framework of a clinical example based on 62 fields of radiotherapy for lung cancer. The delivered doses in monitor units were calculated using three different dose calculation methods: the reference method accounts the dose without tissues density corrections using Pencil Beam Convolution (PBC) algorithm, whereas new methods calculate the dose with tissues density correction for 1D and 3D using Modified Batho (MB) method and Equivalent Tissue air ratio (ETAR) method, respectively. The normality of the data and the homogeneity of variance between groups were tested using Shapiro-Wilks and Levene test, respectively, then non-parametric statistical tests were performed. Specifically, the dose means estimated by the different calculation methods were compared using Friedman’s test and Wilcoxon signed-rank test. In addition, the correlation between the doses calculated by the three methods was assessed using Spearman’s rank and Kendall’s rank tests.

**Results:**

The Friedman’s test showed a significant effect on the calculation method for the delivered dose of lung cancer patients (p <0.001). The density correction methods yielded to lower doses as compared to PBC by on average (−5 ± 4.4 SD) for MB and (−4.7 ± 5 SD) for ETAR. Post-hoc Wilcoxon signed-rank test of paired comparisons indicated that the delivered dose was significantly reduced using density-corrected methods as compared to the reference method. Spearman’s and Kendall’s rank tests indicated a positive correlation between the doses calculated with the different methods.

**Conclusion:**

This paper illustrates and justifies the use of statistical tests and graphical representations for dosimetric comparisons in radiotherapy. The statistical analysis shows the significance of dose differences resulting from two or more techniques in radiotherapy.

## Background

### Radiotherapy techniques

The main challenge in radiation therapy for cancer treatment is to obtain the highest probability of tumor control or cure with the least amount of morbidity and toxicity to normal surrounding tissues (organs at risk). Currently, numerous different machines and several techniques are used to irradiate the tumors, as three–dimensional radiation therapy (3D-RT), intensity-modulated radiation therapy (IMRT), tomotherapy, particle therapy and volumetric-modulated arc therapy (VMAT). The expected clinical results of radiotherapy are related to the calculated dose. The advance in technology provides successive generations of Treatment Planning Systems (TPS) for radiotherapy which include new dose calculation algorithms and allow new irradiation techniques. These algorithms compute the dose for a given technique, subsequently showing the results as dosimetric parameters and displaying dose volume histograms or spatial isodoses. The validation of the treatment plan in radiotherapy is based on the assessment of these TPS output. When a new technique or a new algorithm is implemented, the calculated and distributed doses can differ from those computed by algorithms that constitute the current standard and reference. It is very easy to compare the spatial dose distribution of two DICOM RT files (the file produced by the reference method and the file produced by the new method) by using γ index or χ index [1, 2]. Any difference between the computed dose will be visualized using γ or χ maps [3]. If ignored, this alteration could endanger the clinical outcome of the treatment. In particular, the heterogeneity correction introduces an under dosage to the target when the latter is imbedded in low density tissues, as in thoracic situation. Therefore, prescription habits should be adapted to the new calculation methods and strong connection between dosimetric methods and prescription understanding should be established to avoid dosimetric improvement to turn into clinical regression [4]. Currently, algorithms and irradiation techniques are both able to modify independently the dosimetric outcome. Although the medical physicists validate any treatment plan according to the international recommendations for radiotherapy, these alterations are supposed to have consequences on the clinical outcome [5, 6]. One should provide the radiation oncologist with a tool allowing him to assess the reality and the extent of these modifications, to help him integrate them into his everyday practice. Ideally, the assessment should be possible using small data sets of patients. That way, each department could perform the assessment tests without investing too much time and resources. At this extend, radiotherapy offers the valuable situation in which each patient case could be recalculated in many different ways easily providing paired series of data. This paper presents a series of statistical tests implemented in a step by step procedure that should help the radiation oncologist comparing the dosimetric outcome of different dose calculation algorithms. The procedure is presented using a concrete example and putting the emphasis on the application in radiotherapy rather than the underlying mathematical principles, which are not detailed. Each section gives a brief description of the aim and the condition of use of the statistical tests. The goal is to provide the radiation oncologist and the physicists interpretable results to help them to validate new dose calculation methods.

### The need of statistical analysis in radiotherapy

When comparing algorithms or techniques, the statistical analysis has to answer the key question: are there real differences between two or more calculated dose distributions? By principle, the main objective of any progress in radiotherapy is to improve the clinical outcome by increasing the dose to the tumor and reducing the dose to the organs at risk. The dosimetric progress, by the improvement of the accuracy of the calculation, is supposed to contribute to this goal. Statistical tests are used to make probability-based decisions regarding differences measured between different conditions, e.g., different radiation techniques. For example, are there real dosimetric differences, assessed by the physicist, which could be anticipated by the radiation oncologist as a real benefit or a possible risk for the patient outcome using RT-3D or IMRT techniques.

To compare different techniques or dose calculation algorithms, all dosimetric data are calculated with a unique set of images for a given patient, whatever the number of different algorithms to compare. This provides the radiotherapist with paired data that can be analyzed using statistical tests for repeated measures, e.g., paired t-test when only comparing two techniques or ANOVA when comparing more than two techniques. All statistical tests used in this study were performed with the R programming language [7, 8].

## Methods

### Data collection

The first step consists in preparing and arranging data for further analysis. In order to evaluate the treatment plan for radiotherapy, several dosimetric parameters can be considered:

- Delivered dose in monitor unit.
- Spatial distribution of dose: for example the isodose curves 100% and 95% inside the PTVs can be compared.
- Volumetric distribution: the use of dose volume histogram allows comparing the maximum dose, minimum dose, mean dose, etc.
- Quality index: to compare the conformity plan, the dose homogeneity in PTV and the protection of organs at risk.

Using 3 techniques, one will therefore have 3 values for each dosimetric parameter. In this study, one reference method was compared with 2 new tested methods. Specifically, the reference dose (Dr,1) was compared to (D1,1), and (D2,1). Table 1 presents the data from patients with a sample size “n” using 3 techniques for radiation therapy. The mean dose μ and its standard deviation SD are presented for each technique (μ ± SD).

**Table 1:** **Data collection table for statistical analysis**

Patients	Reference	Tested method 1	Tested method 2
1	D_r,1_	D_1,1_	D_2,1_
2	D_r,2_	D_1,2_	D_2,2_
n	D_r,n_	D_1,n_	D_2,n_
	μ_r_ ± SDr	μ_1_ ± SD_1_	μ_2_ ± SD_2_

For each parameter, data can then be split into two groups: reference data and calculated data resulting from the novel methods. A first simple and very straightforward step consists in assessing whether the dose that would be delivered using the novel method(s) is higher or lower than the one that would be delivered using the reference method. The difference in percent between the novel and reference methods can be calculated as:
1

A positive value means that the dosescalculated by the tested method are higher than the doses calculated by the reference method (D_tested_ > D_reference_). A negative value would mean the opposite (D_tested_ < D_reference_).

For the radiation oncologist to be able to reach the best medical decision it would be valuable to know or be able to estimate how many cases or beams are required to detect potential differences between methods. One way to estimate the number of cases or beams which would be necessary to be able to observe a significant difference between two methods consists in using a power test. For this purpose, the following equation can be used:
2

Assuming that α = 5% the Zα = 1.96 shows the critical value for 95% confidence level for normal distribution.

To determine the sample size, one should beforehand, set the significance level (α) and the statistical power of the test to come. α defines the probability of “erroneously” concluding that the observed difference between means reflects a real difference between methods when this difference between means was actually observed by chance. α is typically set to 0.05 (p-value) which corresponds to a 5% chance to conclude to a significant difference when there is no actual difference. Statistical power corresponds to the probability of detecting an actual difference. Applied to the comparison of radiotherapy methods, statistical power therefore corresponds to the probability of “correctly” concluding that the observed difference between means reflects a real difference between methods. A conventional choice of power is either 80% or 90% [9, 10]. Power is usually calculated before data collection in order to estimate the required sample size. In some case, statistical power is calculated after data collection to verify whether the non-significance of the result might be due to a lack of power, but this practice is usually discouraged [11]. Here we calculated statistical power after data collection for illustrative purposes. For that, we used PS software, which provides estimates of power and sample size, and allowed us to plot sample size versus power for a specified alternative hypothesis [12].

In addition to this power calculation, we used bootstrapping to estimate the minimum number of patients or cases that we would have needed to observe a significant difference between methods or groups with our data. We chose this a posteriori approach because we already had data collected for 62 beams. It also allowed us to compare the results with the estimation provided by the power analysis.

As an additional note, we would like to mention that P-values do not really measure the importance of the effect. Therefore, statistical tests can be complemented by an assessment of the effect size (effsize). We choose to use “Pearson’s r”, to measure and estimate the amount of total variance in the data resulting from the difference between the compared groups.

### Comparing methods

Several statistical tests can be used to compare means. These tests can be classified in two categories: parametric and non-parametric tests. Parametric tests make assumptions about the type and distribution of the data. If the data fulfill the assumptions of parametric data, parametric tests can be used. If the data do not fulfill these assumptions, non-parametric tests must be used. Non-parametric tests make fewer assumptions about the type and distribution of the data.

### Checking assumptions of parametric data

**Figure 1:**
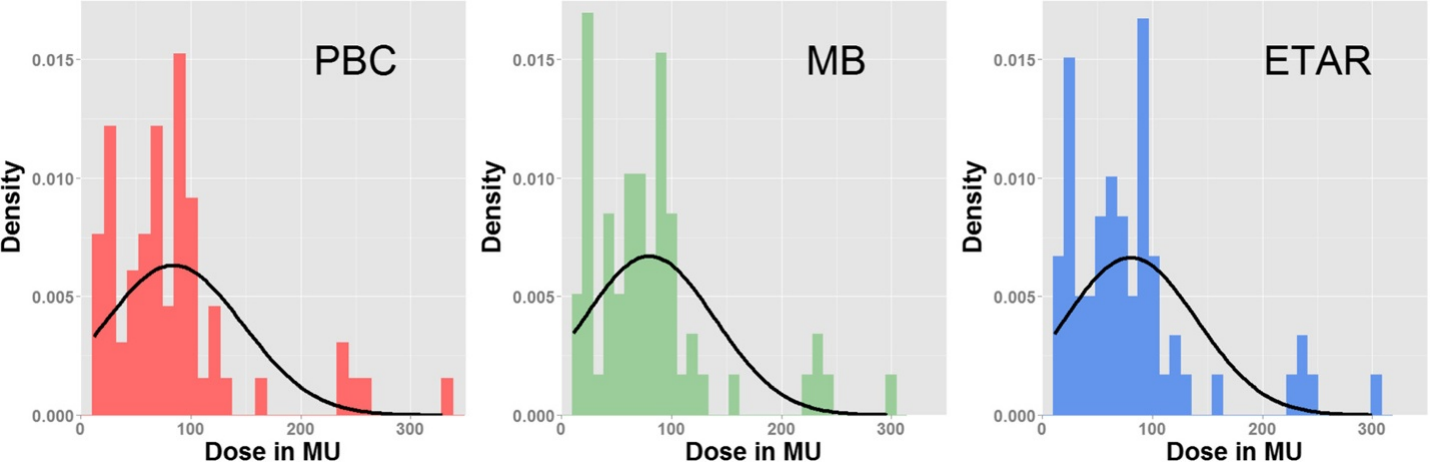
**Density plots showing the deviation of normality and the positive skewness (i.e., clustering at the lower end (left) of the scale).** Negative skewness would have shown clustering at the higher end of the scale. A normal curve using the mean and standard deviation of the data as parameters has been drawn on top of the histograms to show the deviation of normality.

Before choosing a method to compare means, two main assumptions regarding data distribution must be assessed. Specifically, one should test whether: data are normally distributed and the variances of the samples to be compared are similar (homogeneity of variance). Several statistical tests can be used to assess whether data are normally distributed or not (see [13] for an overview). Among those, the test that is the most widely used is the Shapiro-Wilks test, notably because it is more powerful than most other normality tests [14], i.e., it provides higher probability of detecting actual departure from normality in the data (statistical power is addressed above). The Shapiro-Wilks test estimates the probability that any given sample is drawn from a normal population. It can be complemented by computing the skewness and the kurtosis of the sample distribution. The skewness relates to the symmetry of the distribution (as shown in Figure 1). A skewed distribution is not symmetrical as the most frequent scores are clustered at one end of the scale. Kurtosis gives the degree to which scores cluster at the end of the distribution. A normal distribution has both a skewness and an excess kurtosis of 0. To assess whether the skewness and kurtosis of a sample deviate significantly from those of a normal distribution, one can divide the calculated skewness and kurtosis values by their standard error. Resulting absolute values greater than 1.96 indicates a significant deviation. These statistical tests should always be complemented by a visual inspection of the data.

Density distribution histograms are very useful for assessing normality. This type of representation displays the density distribution of the scores. In other words, it provides visual information about the shape of the distribution. In the ideal case, the data should be distributed symmetrically around the center of all scores. The data distribution can deviate from normal distribution if there is a lack of symmetry (skewness). Skewed distributions are not symmetrical and, instead, the most frequent scores (the tall bars on the graph) are clustered at one end of the scale. So, the typical pattern is a cluster of frequent scores at one end of the scale and the frequency of scores tailing off towards the other end of the scale. A skewed distribution can be either positive or negative according to the shape of the asymmetry.

**Figure 2:**
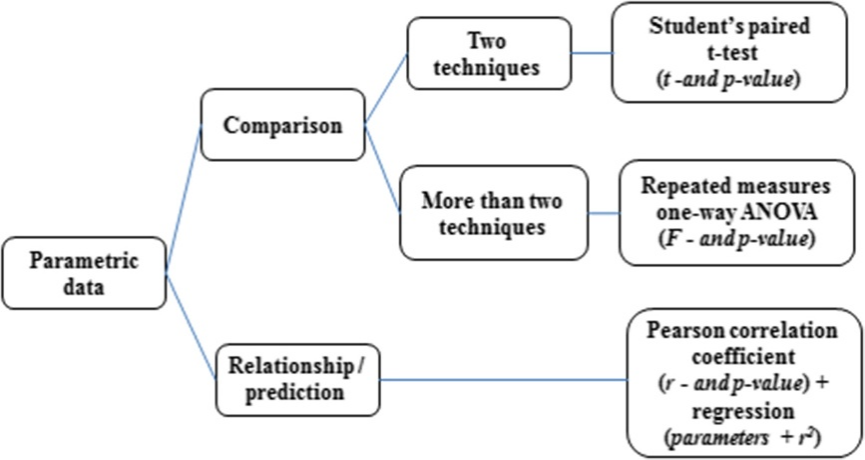
**Flowchart of statistical methods for parametric data.**

**Figure 3:**
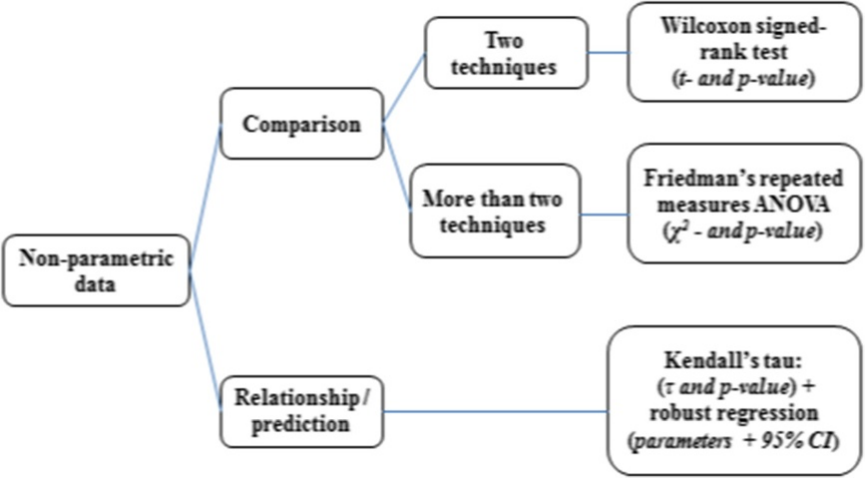
**Flowchart of statistical methods for non-parametric data.**

To assess whether the assumption of homogeneity of variance is fulfilled, the most commonly used test is the Levene’s test. However, the Brown-Forsythe test constitutes a more robust (and often preferable) alternative. These tests estimate whether the variance is similar/comparable in the different samples by analyzing deviations from the mean (Levene) or the median (Brown-Forsythe) of the distribution. Applied to our radiotherapy example, these tests can be used to assess whether the variance of the dosimetric values calculated by the different techniques is similar. One should mention that testing for homogeneity of variance is not necessary with repeated measures design, i.e., designs in which the same subjects are tested on the different conditions (as it is the case here). However, we mention it in the analysis for the sake of the example. Box and whisker diagram boxplot is a type of visual representation that completes the Levene’s or Brown-Forsythe test when evaluating the homogeneity of variance between groups. Boxplots provide important information about the distribution and variability of the data. They display the minimum and maximum values of the distribution as well as the 25th percentile, 50th percentile (median) and 75th percentile. Figures 2 and 3 show the statistical methods which could be used for radiotherapy depending on whether data are parametric or not.

### Comparison of techniques

To compare two techniques (i.e., two means) when measures are repeated, paired Student parametric test or Wilcoxon signed-rank non-parametric test can be used, as shown in Figures 2 and 3. With independent measures (i.e., independent samples), independent Student t-test and Mann Whitney test for non-parametric data are usually used.

To compare more than two techniques, a two-step analysis should be conducted. The first step consists in conducting an omnibus test to assess whether there is an overall effect of technique, i.e., whether all techniques are the same or not. If the data fill the assumptions of parametric data (normally distributed data and homogeneity of variance between groups), this omnibus test can be performed using a one-way Analysis of Variance (ANOVA) for repeated measures, with correction for violation of sphericity if needed [15]. To compare the outcome of different techniques for more than one dosimetric parameter, one can use a multivariate analysis of variance (MANOVA), but this point will not be discussed here. If the data do not fill the assumptions of parametric data, the omnibus test must be performed using a non-parametric equivalent of the repeated measures ANOVA, namely Friedman’s repeated measures test (the Kruskal-Wallis test can be used for independent measures). Once this omnibus test has been performed, and if a main effect has been detected, the second step consists in conducting multiple paired-t-tests (multiple comparisons) to compare techniques in a two by two fashion. With parametric data, these comparisons can be performed using multiple paired t-tests (see above). With non-parametric data, multiple Wilcoxon signed-rank tests can be used. For both parametric and non-parametric multiple comparisons, the significance level must be corrected in order to avoid inflating type-1 error probability, i.e., the probability of detecting a significant difference when actually there is not any (i.e., false positive). One way of doing that is to divide the significance threshold by the total number of paired comparisons performed (e.g., with three comparisons, alpha = 0.05/3 = 0.0166). This correction method is known as Bonferroni correction. Bonferroni correction is the most conservative correction method. Specifically, it controls strictly the probability of false positive, but this is at the cost of reducing the statistical power of the test, i.e., the ability to detect an actual difference. When power is an issue, less conservative correction methods can be used, as for instance the Holm method, in which the correction factor applied depends not only on the number of comparisons performed but also on the rank of the p values. Finally, several other post-hoc procedures are available to compare several means, as Tukey’s honest significant difference test (HSD), Newman-Keuls test, or Fisher’s least significant difference (LSD) test, to cite a few. Tukey’s HSD is one of the most widely used, especially when the number of means to compare is relatively large, as it offers good control over false positives while keeping decent power (see [16] for a specific comparison between post-hoc procedures).

### Correlation between techniques

To measure the strength of the relationship between two techniques (i.e., estimate how much two techniques are related), for parametric data, Pearson correlation coefficient can be used. For non-parametric data, one should rather use Spearman’s ‘rho’ correlation coefficient or Kendall’s ‘tau’. In this study, the data did not fill the assumptions of parametric data. Therefore, Spearman’s rank correlation and Kendall’s rank were used.

### Medical decision

A p-value (p ≥ 0.05) implies that the differences observed between means probably occurred by chance (rather than reflecting a real difference between the methods). In this case, we can assume that there is not enough evidence to conclude that the new technique gives rise to significant dosimetric difference with respect to the reference technique. Conversely, a p < 0.05 implies that the differences observed probably reflect existing differences between the methods. In this case, we could expect a significant dosimetric difference and therefore some medical impact.

### Clinical tests for radiotherapy

The following analyses of data from radiotherapy present an overview of how statistical methods may be used for the evaluation of real treatment plans. In this study, a very common improvement of dose calculation was used as an example of a software change producing a true situation of decision in radiotherapy. The statically methods described in this study have been applied to lung cancer using 62 treatment fields with 18MV photon beams. Table 2 shows the site locations, the prescribed dose and the treatment fields for all patients.

**Table 2:** **Characteristics of the patients treated with 3D radiotherapy using 18MV, location tumour, dose prescription and treatment fields**

Patients	Locations	Dose (Gy)	Fields
1	Lung parenchyma	66	6
2	Left retro cardiac	66	17
3	Top left lung	70	10
4	Mediastina	60	9
5	Mediastina	60	12
6	Oesophagus	55	8

The aim was to compare the delivered dose in monitor units resulting from three dose calculation methods keeping exactly the same beam setting. Calculations dose were performed, for the present demonstration, using the Pencil Beam Convolution (PBC) algorithm integrated in TPS Eclipse® (Varian, version 8.1). PBC algorithm includes two calculation modes: without heterogeneity correction using (PBC) and with heterogeneity correction using the Modified Batho (MB) method and Equivalent Tissue air ratio (ETAR) method. Heterogeneity corrections are always based on relative electron densities obtained from a CT-scan [17, 18].

Reference method: calculates the dose without taking into account the tissues densities using PBC.

MB method: this method is based on the Tissue Maximum Ratio (TMR) and calculates the density distribution in 1D. The correction factor is given by:
3

Where μ_m_ and μ_w_ are the linear attenuation coefficients of the material in layer (m) and water (w) respectively; Z_bu_ is the build-up depth and Z_m_ is the distance along the beam from the surface to the layer (m) in the phantom. μ_en_/ρ is the mass energy absorption coefficient of the material in layer (N).

ETAR method: calculates the dose taking into account 3D tissues densities correction. It uses the Tissue Air Ratio (TAR) dependent on the effective beam radius () to take account of scattered radiation and effective depth (*d*′) for primary beam correction. The correction factor is given by:
4

Where  are the effective values of depth (d) and beam radius (r) respectively.

The statistical analyses in this study were performed to assess whether using new method MB or ETAR rather than the reference method (PBC) would give rise to a significant reduction or increase in the delivered dose. The analyses were carried out using the R environment for statistical computing and visualization. All data were imported in R from Excel®.

## Results

### Assessment of the assumptions of parametric data

#### Normality

Figure 1 shows the density distribution of the dosimetric values calculated by the three methods. A clear deviation from normality and positive skewness (i.e., clustering at the lower end (left) of the scale) can be observed. Table 3 presents the results of the Shapiro-Wilk test, as well as skewness and kurtosis values (divided by their standard error) for the reference and two new methods. The Shapiro-Wilk test shows a significant deviation from the normality (confirming what can be visually observed in Figure 1). We note also a significant positive skewness and significant kurtosis p < 0.05.

**Table 3:** **Observed results from Shapiro-Wilks test, skewness and excess kurtosis for the reference and two new methods**

Tests	Results	Reference	Method 1	Method 2
Shapiro-Wilks	W	0.80	0.81	0.81
	p-value	<0.001	< 0.001	< 0.001
Skewness	Skewness/standard error	6.05	5.64	5.68
	p-value	< 0.001	< 0.001	< 0.001
Excess Kurtosis	Kurtosis/standard error	6.15	5.04	5.14
	p -value	< 0.001	< 0.001	< 0.001

#### Homogeneity of variance

**Figure 4:**
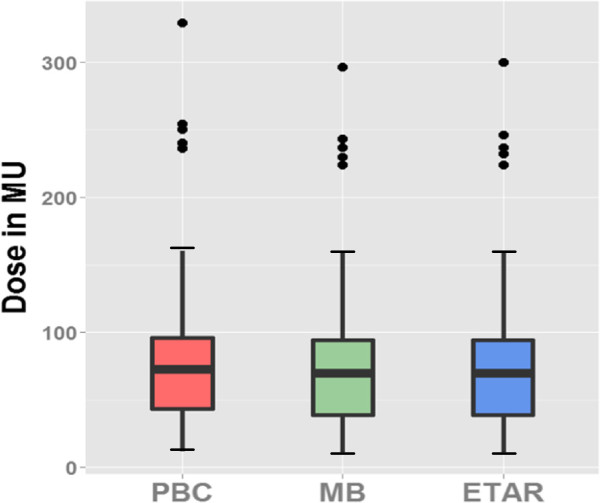
**Boxplot indicating the minimum and maximum values, the 25th percentile, 50th percentile (median) and 75th percentile.**

Figure 4 shows the boxplot for all three methods indicating 62 fields. Because we are dealing here with repeated measures, running a Levene’s or Brown-Forsythe test is not necessary. We ran nonetheless a Brown-Forsythe test for the sake of the example. It indicates that the different groups have significantly different variances, F(2,183) = 0.987 and p < 0.05, even if this difference is not striking when examining boxplot representations of the three distributions (Figure 4).

All diagnostic tests indicate that the data do not fulfill the assumptions of parametric data. Accordingly, statistical tests were conducted using non-parametric methods. As presented in the Figure 3, means were compared using the Friedman rank and Wilcoxon signed-rank tests.

### Comparisons of dose calculation methods

**Figure 5:**
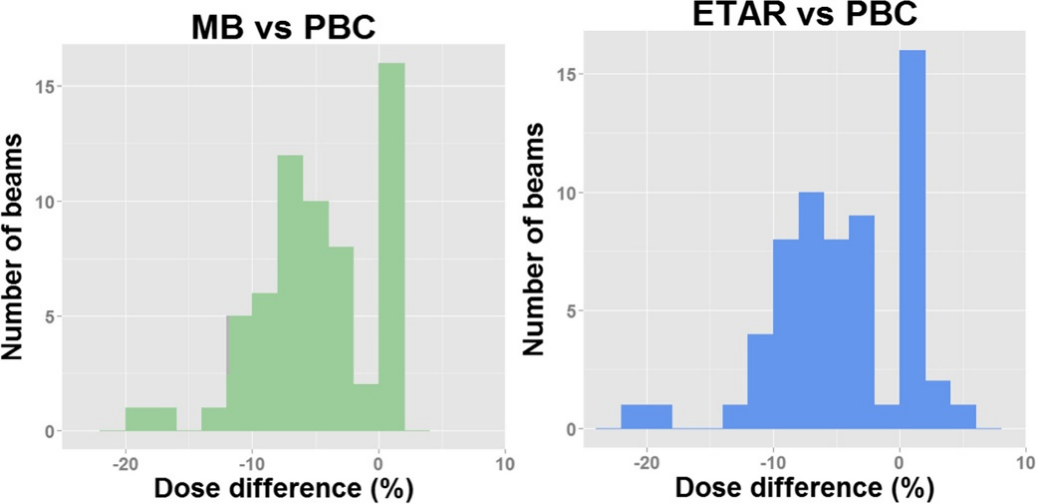
**Frequency distribution of dose differences between PBC with MB (left) and ETAR (right).** For the majority of beams, the calculated dose is lower with MB and ETAR than with PBC.

Figure 5 shows the number of beam distributions regarding the dose differences (in percent) between the reference method and new methods 1 and 2. The density correction methods yielded to lower doses as compared to PBC by on average (−5 ± 4.4SD) for MB and (−4.7 ± 5SD) for ETAR. Considering these results, the use of MB or ETAR instead of PBC to treat the patients would result in under dosage in the PTVs. This difference may have a clinical impact compared to the reference plan.

Therefore, one should assess whether this difference is significant or rather occurred by chance. Friedman rank sum test showed a significant overall effect of technique,χ^2^(2) = 53.45, p < 0.001.

**Figure 6:**
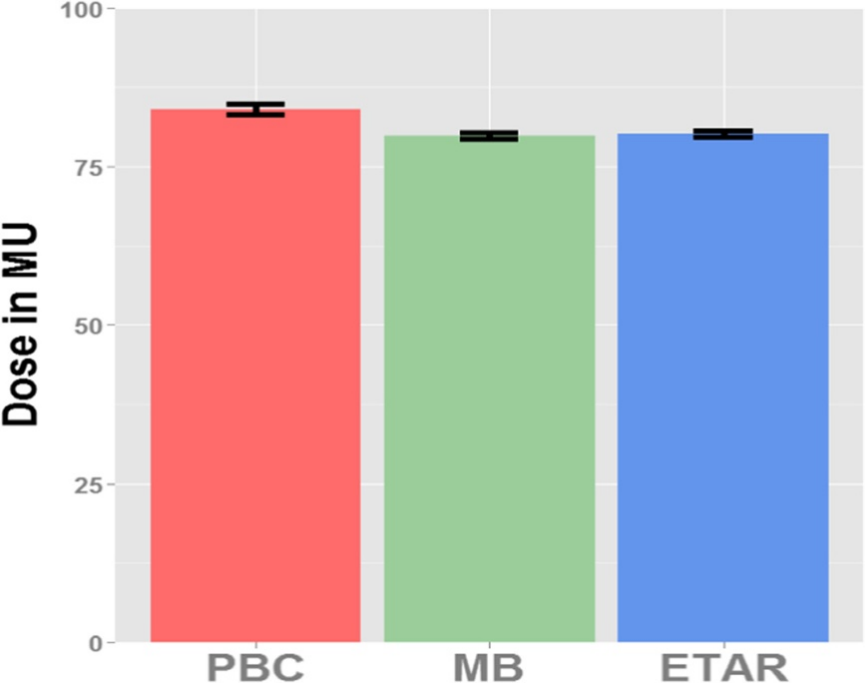
**The bars show the mean dose that would be delivered using the different methods.** Error bars indicate the 95% confidence interval adjusted for repeated-measures.

MB and ETAR methods were then individually compared to PBC using Wilcoxon signed-rank tests. The tests showed that both methods gave rise to doses that were significantly different from the PBC, sum all positive and negative ranks V_Wilcoxon_ =1207 and 1216 for MB vs PBC, and ETAR vs PBC, respectively, with p < 0.001 in both cases. The “Pearson’s r” test showed effsize = −0.53 and −0.50, for MB vs PBC and ETAR vs PBC, respectively. On the other hand, MB and ETAR did not differ from one another, V_wilcoxon_ = 121.5 with p = 0.31 (which is far above the corrected “p” for multiple paired-comparisons = 0.05/3 = 0.016) and effsize = −0.19. Figure 6 presents the mean dose that would be delivered using PBC, MB and ETAR. For each method, error bars indicate the 95% confidence interval adjusted for repeated-measures according to Loftus et al. [19].

**Figure 7:**
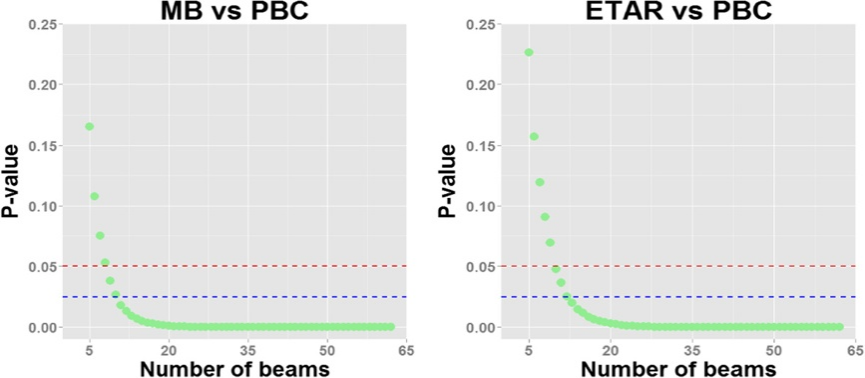
**p-values estimated by bootstrap procedure, indicating the average p-value for each sample-sizes going from 5 to 62.** The left panel corresponds to the comparison between MB and PBC, and right panel to the comparison between ETAR and PBC. The red dashed line corresponds to a significance threshold of 0.05 and the blue dashed line to an adjusted significance threshold of 0.025 (0.05/2 to correct for two comparisons).

As reported above, with 62 beams, we observed significant differences between MB and PBC as well as between ETAR and PBC. In order to determine how many subjects or beams would have been necessary to observe significant differences between our methods (i.e., MB vs PBC and ETAR vs PBC), we used a bootstrap procedure. This consisted in taking 1000 random samples of size “n” (with n going from 5 to 62) for each of the two methods MB and ETAR we wanted to compare. For each sample, p-value was computed using Wilcoxon signed-rank test. For every “n” the mean p-value across the 1000 random samples was computed. Figure 7 shows the computed mean p-values for each sample size (from 5 to 62 beams). As shown in the left panel, 8 beams would have been sufficient to observe a significant difference between MB and PBC, whereas observing a significant difference between ETAR and PBC would have required 10 beams (right panel).

**Figure 8:**
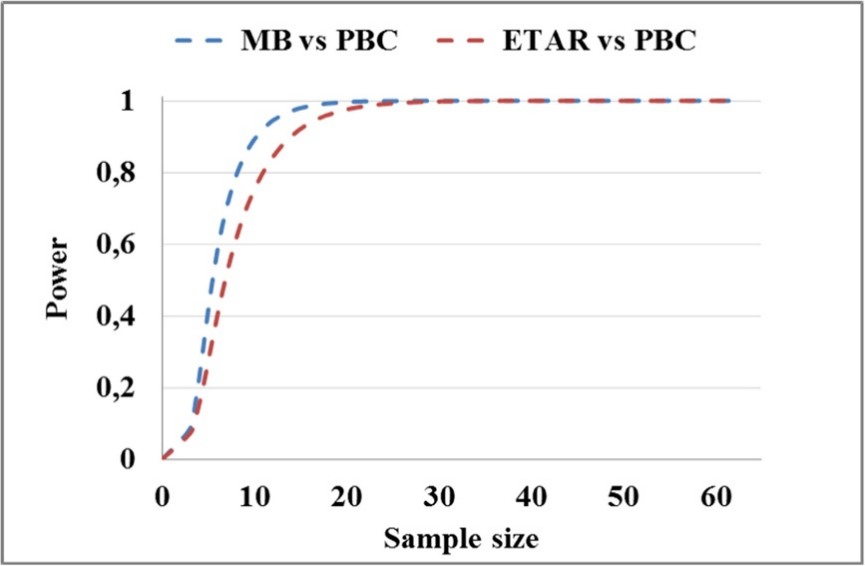
**Statistical power as a function of sample size using Wilcoxon test.**

Figure 8 shows the statistical power as a function of sample size using Wilcoxon signed-rank test. It can be seen from Figure 8 that to achieve 80% power it requires about 8 and 10 treatment fields to compare PBC with MB and ETAR, respectively. These estimations correspond well to the numbers provided by the bootstrap procedure reported above. We can also note that the use of 62 treatment fields in this study leads a very high power.

### Relationship between dose calculation methods using correlation and regression

**Figure 9:**
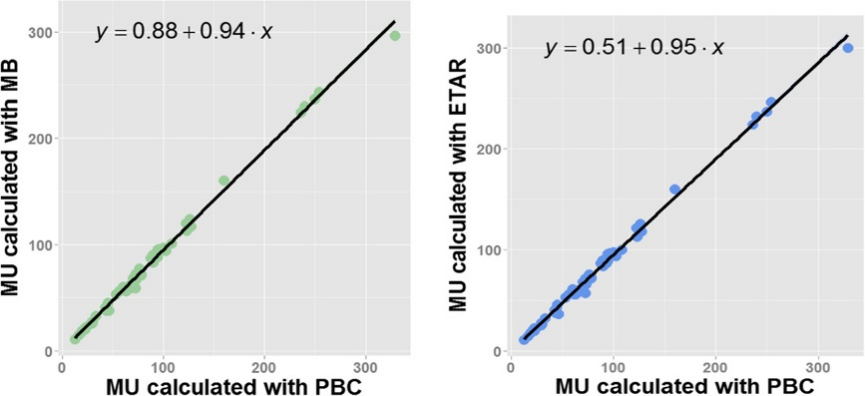
**Correlation between PBC with MB (left) and ETAR (right).** The line is computed using a least square regression method, and is defined by its slope b_1_ and intercept b_0_ as: Y_i_ = b_0_ + b_1_ *X*_*i*_, where Y_i_ is the outcome, *X*_*i*_ is the *i* ^th^ participant’s score of the predictor variable.

Figure 9 shows the correlation plots between the reference and the new methods. It can be seen in Figure 9 that there was a strong correlation between PBC with MB and ETAR. Specifically, for MB, Kendall’s rank ‘tau’ was 0.95 (95% BCa (confidence interval using bootstrapping) [0.91; 0.97]) with p < 0.001, and Spearman’s rank ‘rho’ was 0.99 (95% BCa [0.99; 1.0]) with p < 0.001. For ETAR, Kendall’s rank ‘tau’ was 0.94 (95% BCa [0.90, 0.96]) with p < 0.001, and Spearman’s rank ‘rho’ was 0.99 (95% BCa [0.98, 1.0]) with p < 0.001.

The 95% BCa for the slope was [0.90 ; 0.96] and [0.92; 0.97] for the comparison between PBC with MB and ETAR respectively. The 95% BCa for the intercept was [−0.83 ; 3.67] and [−1.14 ; 2.95] for the comparison between PBC with MB and ETAR respectively. In both cases, the coefficient of determination was very high (R^2^ = 0.996) with p < 0.001. The 95% confidence bootstrap percentile (95% BCa) interval for correlation and regression has been computed using non-parametric bootstrapping with 2000 replicates.

### Medical decision

The mean comparison tests between the PBC method with MB or ETAR indicated significant differences in dose calculation (p < 0.001). In other words, the observed differences probably reflect existing differences between the methods. In addition, the bootstrapping procedure indicated that significant differences between the reference and the new methods could be observed with as little as 8 and 10 beams, respectively. Therefore, the observed dosimetric differences between methods very likely bear a clinical impact.

The regression analyses show slopes of 0.94 and 0.95 for MB and ETAR, respectively (see Figure 9). The slope represents the change associated with the MB and ETAR compared to PBC. Should these methods be equivalent, these values for slope would have been equal to one. In this study, the slope values are below 1, with 95% confidence intervals of [0.90; 0.96] and [0.92; 0.97], respectively. These slope values confirm the average difference of 5% and 4.7% computed with equation 1 for MB and ETAR. This overall difference is the main result of this study, and confirms that the prescribed dose should be adjusted by on average +5% and +4.7% using MB and ETAR, respectively.

However, different cancer sites should be considered individually before making any general rule of dose modification. We recommend presenting the complete set of statistical information including mean, SD, confidence intervals, p-value, sample size and graphical data analysis to the medical staff. These information will help the radiation-oncologists to take a decision about the modification of the irradiation technique and dose prescription. The confidence interval, which is routinely computed by all statistical packages, shows the size of difference which could be observed. The width of a confidence interval depends on the mean and standard deviation of the results.

## Discussion

Among the numerous statistical methods available, the medical physicist has to make a choice well adapted to the particularities of radiation therapy. At first, the particular nature of the data and the way they are produced need a deep analysis of their quality. In particular, one should assess whether the data at hand fulfill the assumptions of parametric data, i.e., are distributed normally and have similar variance between groups. When the data fulfill these assumptions, Student t-test or one way ANOVA can be used to compare means. But if data do not fulfill these assumptions, alternative non-parametric tests should be used, as for instance the Wilcoxon signed-rank test or Friedman ANOVA (when dealing with repeated measures). The non-parametric Wilcoxon rank test takes into account the signed-rank of the difference between each pair of measures instead of using all the absolute data. It does not require a normal distribution and does not considers the size of the difference. These features make the Wilcoxon signed-rank test particularly fit for radiotherapy data analysis, since these data are “naturally” paired according to the possibility to generate multiple different results with each medical case. In case of simultaneous comparison of multiple parameters (i.e., not only the delivered doses per monitor unit but also volumetric distribution, etc.), multivariate ANOVA can be performed (not used in this paper). However, an in-depth discussion between medical physicists and statisticians is strongly encouraged in order to determine which tests and analyses are the most appropriate for the data at hand (e.g., use of parametric vs non-parametric tests).

We compared one parameter using three methods for calculation dose. The example given in this study concerning the delivered dose using 1D or 3D dose calculation is an exemplary problem in radiotherapy. The clinical effect in radiotherapy depends on the delivered dose, a small difference in delivered dose being worth to be considered since it could affect the clinical results. Concerning the delivered dose, we observed a significant difference between reference method and both tested methods (p < 0.05). However, p-value gives little information about the results, for example, using 5% level for alpha error, the p-value shows only if the difference between the two techniques has a statistical significance or not. It does not give the size of the difference between two techniques. This information can be gathered computing the percentage of difference between two techniques (as we did here) but also computing the effect size (here the Pearson’s r) which is a standardized measure (always between −1 and 1) of the importance of the observed effect. In our study, the measured effect sizes were quite large (above 0.5 in absolute value) for both comparisons of the new methods with the reference method.

The calculation of sample size is crucial in any clinical study. It depends on the significance level, the wished power, as well as the expected effect size and SD. The sample size for clinical and statistical studies is the main difficulty for radiotherapy. For practical reasons, it would be welcome to use only few patients for realizing the statistical analysis, and then to generalize the results to a large population. In radiotherapy it is rare to use a large number of patients in order to validate the novel irradiation technique at the level of a common department. Most of the studies in radiotherapy are including between 10 and a few hundred patients. Most of the time, a novel technique is quickly integrated in a radiotherapy department and the physicists has not enough time to test a large sample of patient. In this case, the radiation-oncologist and the physicists will have to realize the statistical analysis using a small sample size, which is actually possible providing one uses cautiously the proper methods. As mentioned above, one possibility to estimate the required sample size for an expected effect size is to use a power analysis. Because we already had data collected (62 beams), we chose to rather use a bootstrap approach to estimate the minimum number of patients or cases that we would have needed to observe a significance difference between methods or groups. We have seen that a significant difference between our methods could be observed with relatively small sample, namely eight and ten beams.

Interestingly enough, this approach is basically funded on the analysis of differences and in particular dose distribution differences. Therefore it can be adapted to compare any type of situation resulting at last in dosimetric differences as different irradiation methods, different machines, even assessing the identity of mirror machines, and so on.

## Conclusion

We illustrate in this study the use of statistical tests for paired observations well adapted to the specificity of radiotherapy where different data sets can be obtained of the same patient. To compare two radiotherapy techniques, the standard t-test can be used with parametric data, whereas non-parametric Wilcoxon signed-rank test should be used if the dose differences do not fulfill the assumptions of parametric data. To compare several techniques, the standard analysis of variance (ANOVA) can be used with parametric data, whereas non-parametric tests as the Friedman ANOVA should be used with non-parametric data. Here we suggested statistical methods to compare calculated doses in radiotherapy. These methods can also be used to compare measured doses, e.g., using two or more dosimeters, as well as to compare calculated and measured doses.
